# A Brief History of Airway Smooth Muscle's Role in Airway Hyperresponsiveness

**DOI:** 10.1155/2012/768982

**Published:** 2012-10-18

**Authors:** C. D. Pascoe, L. Wang, H. T. Syyong, P. D. Paré

**Affiliations:** ^1^James Hogg Research Center, St. Paul's Hospital Vancouver, University of British Columbia, Vancouver, BC, Canada V6Z 1Y6; ^2^Respiratory Division, Department of Medicine, University of British Columbia, Vancouver, BC, Canada V5Z 1Mg

## Abstract

A link between airway smooth muscle (ASM) and airway hyperresponsiveness (AHR) in asthma was first postulated in the midnineteenth century, and the suspected link has garnered ever increasing interest over the years. AHR is characterized by excessive narrowing of airways in response to nonspecific stimuli, and it is the ASM that drives this narrowing. The stimuli that can be used to demonstrate AHR vary widely, as do the potential mechanisms by which phenotypic changes in ASM or nonmuscle factors can contribute to AHR. In this paper, we review the history of research on airway smooth muscle's role in airway hyperresponsiveness. This research has ranged from analyzing the quantity of ASM in the airways to testing for alterations in the plastic behavior of smooth muscle, which distinguishes it from skeletal and cardiac muscles. This long history of research and the continued interest in this topic mean that the precise role of ASM in airway responsiveness remains elusive, which makes it a pertinent topic for this collection of articles.

## 1. Introduction

**Figure 1:**
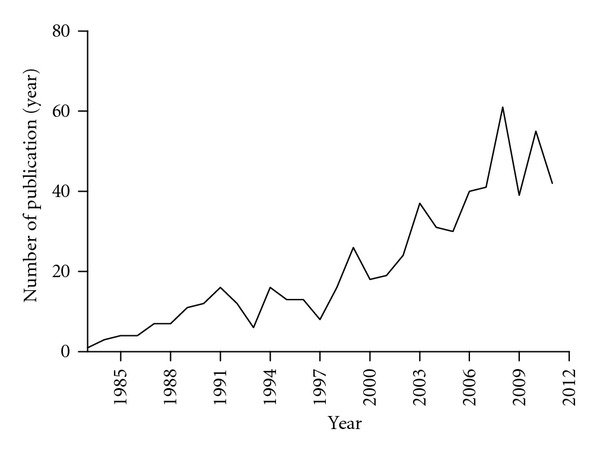
PuMed search for (ASM + AHR + asthma).

In this paper we review the history of the link between airway smooth muscle (ASM) and the phenomenon of bronchial hyperreactivity or hyperresponsiveness (BHR) which is a defining feature of asthma.  Figure 1 shows the number of PubMed citations generated by a search for “airway smooth muscle + airway hyperresponsiveness + asthma.” These results suggest that there is an increasing interest in the role of ASM in AHR, but what is the history of evidence to support a link?

It has long been recognized that muscular constriction of the bronchi contributes to airway narrowing in asthma. In his 1698 treatise on asthma Floyer wrote, “the Bronchia are contracted … and that produces the Wheezing noise in Expiration, and that this Symptom does not depend on Phlegm is plain, because the Hysteric, who have no Phlegm, Wheeze very much” [1]. In mid-nineteenth century, Salter [2] wished that it could be “shown beyond cavil that spasmodic stricture of the bronchial tubes is the only possible cause of asthma, that it is adequate to the production of all the phenomena.” He was referring to the “spastic contraction of the fiber-cells of organic muscle,” which we now refer to as the airway smooth muscle (ASM). 

In a landmark study of the pathology of asthma Huber, and Koessler [3] described and quantified the increased mass of ASM. The accumulated evidence for an increase in muscle mass and the relative contributions of hypertrophy and hyperplasia to this process has been recently summarized [4]. Thus there is little doubt that ASM is increased in asthma. The questions that remain are whether this increase is the cause of airway hyperresponsiveness (AHR) or whether there are additional fundamental changes in the phenotype of the muscle which contribute to AHR. 

 Bronchial responsiveness in asthmatics was first reported by Alexander and Paddock in 1921 [5] when they noted that an attack could be precipitated by subcutaneous injections of pilocarpine. Similarly Weiss et al. [6] found that asthmatics became more breathless and had a greater fall in vital capacity in response to intravenous histamine than did non-asthmatic subjects. Subsequent early studies confirmed that asthmatics responded excessively to a wide variety of stimuli including acetyl-beta-methylcholine [7], carbachol [8], histamine [9], slow reacting substance of anaphylaxis [10], prostaglandin F2*α* [11], propranolol [12], cold air [13], sulphur dioxide [14], dust [15], and exercise [16]. Most of these act directly on smooth muscle but others act to cause secondary release of contractile agonists (e.g. cold air) or inhibition of bronchodilating factors (e.g. propanolol). In more recent literature these methods of eliciting an airway response have been termed *direct* (via ASM) and *indirect* (via release of inflammatory mediators and subsequent ASM activation) measures of airway responsiveness. 

These studies illustrated a key feature of AHR, that it is nonspecific. If a subject is hyperesponsive to one stimulus they are hyperrresponsive to all agents that act by stimulating smooth muscle contraction. This observation was important since it suggested that the phenomenon was primarily postjunctional (i.e., on the muscle side of the neuromuscular junction) and not related to specific abnormalities of any specific agonist receptors on smooth muscle cells). This supports the hypothesis that a change in ASM phenotype is responsible for the phenomenon of AHR in asthma but an additional important early observation was that AHR was not limited to asthmatics. Patients with a variety of diseases characterized by airway obstruction show AHR and the degree of airway responsiveness is related to the degree of baseline airway obstruction [17–19]. These results suggest that the responsiveness may be consequence of the airway narrowing rather than a predisposing factor. However in asthma, AHR is relatively independent of baseline lung function [20] suggesting that the underlying mechanisms may be distinct from the AHR seen in COPD and other airway diseases. 

A final seminal early observation was that there were variations in airway responsiveness over time. De Vries et al. showed that there was diurnal variation in responsiveness [21] with the greatest responsiveness occurring at night when the baseline airway narrowing tends to be greatest. Kerrebijin (1970) showed that AHR increases after an acute spontaneous attack of asthma and then improves over time as the attack subsides [22], again suggesting that AHR is a consequence of asthma, or at least that a portion of the AHR was variable and unlikely to represent a fundamental phenotypic change in the muscle. Parker et al. showed that AHR occurred during or after a respiratory tract infection in normal subjects supporting the concept of acquired, reversible AHR [18]. Additional important observations were that AHR increased after the late, but not the early, asthmatic response [23] to inhaled allergen and that AHR could be attenuated by prolonged anti-inflammatory therapy [24]. 

## 2. Airway Smooth Muscle and Airway Responsiveness

Despite the increasing interest in AHR during the 60s and 70s, there were few attempts to study the mechanism. As early as 1951, Schild et al. [25] found that lung tissue and bronchial muscle obtained from an asthmatic patient released more histamine and responded with contraction to challenge with house dust or pollen compared to a non-asthmatic, but it was not until the early 1980s that there was speculation that AHR was caused by an intrinsic alteration in ASM structure or function. The prevalent theories prior to that where related to pre-existing airway narrowing [26, 27], increased sensitivity of airway irritant receptors [28] or a relative deficiency of beta adrenergic bronchodilation [29]. Freedman [30] and Benson [26] were among the first to systematically consider the potential link between the structural and functional changes in the airways and AHR. They pointed out that airway wall thickening and/or baseline airway smooth muscle tone could amplify the airway narrowing caused by a subsequent stimulus supporting the concept that AHR was a manifestation of airway disease not a root cause. 

A pivotal study by Woolcock et al. [31] published in 1984 showed that an important feature of AHR in asthma was an increase in maximal achievable airway narrowing in response to histamine; most nonasthmatic subjects can inhale high concentrations without much airway narrowing. They showed that asthmatics not only show a response at a much lower dose or concentration than nonasthmatics (increased sensitivity) but that the amount of airway narrowing measured by a decline in forced expiratory flow is much greater. This was attributed to a lack in asthmatics of a normal mechanism that inhibits severe airway narrowing in nonasthmatics and they hinted at a link to maximal ASM contraction.

Studies of excised human airway smooth muscle began in the 1980s and for the most part failed to incriminate ASM, although most of the initial studies examined only isometric force. Although some studies suggested that ASM from asthmatics was stronger [32, 33] the bulk of the data [34–38] show that the maximal force that ASM can generate does not differ in asthmatic and nonasthmatic individuals. These studies spawned a number of different avenues of investigation in an attempt to explain AHR. Generally these studies focused on additional properties of ASM that could be important in generating AHR or on additional explanations for AHR that did not involve a fundamental change in ASM phenotype. In 1986, Moreno et al. [39] presented an extensive theoretical analysis of the geometric factors which could link ASM activation and excessive airway narrowing, amplifying the earlier work of Freedman [30]. James et al. [40] and Wiggs et al. [41] quantified the potential contribution of airway wall remodeling to increased maximal airway narrowing. Lambert et al. [42] concluded that the increase in smooth muscle mass was potentially the most important structural change to explain AHR (provided that the increased muscle mass retained its contractile phenotype). This conclusion has been supported by recent work from Oliver et al. [43] who added cyclical stress to the model to simulate breathing. Their analyses confirmed the importance of increased muscle mass and also suggested that increased muscle could explain the failure of asthmatics to respond to deep inspirations. 

The other mechanical properties of ASM that have been explored as potential contributors to AHR include an increased maximal amount of shortening, increased velocity of shortening, reduced relaxation, and a reduced effect of strain on the reduction of force that occurs with breathing and deep inspiration. Ma et al. [44] examined the maximal shortening and the shortening velocity of primary isolated ASM cells from asthmatic and normal subjects and found both greater maximal shortening and faster shortening associated with an increased expression of myosin light chain kinase. Léguillette et al. [45] studied the relative expression of two isoforms of human myosin in the ASM of asthmatic and nonasthmatic subjects. They found that there was increased mRNA for the SM-B isoform in asthmatic tissue. SM-B contains a 7 amino acid insert and can be shown to propel actin faster than the SM-A isoform. These data suggest that a change in the relative proportion of the two myosin isoforms could increase ASM shorting velocity and could increase AHR in asthmatics. They did not measure the relative abundance of the protein for the two isoforms. 

## 3. Airway Smooth Muscle Adaptability

A whole new area of investigation was heralded in 1995 by the publication by Pratusevich et al. [46] showing that unlike skeletal muscle the length tension relationship of smooth muscle is plastic; the length at which maximal force and shortening occur can change dependent on the length history of the smooth muscle. This observation coupled with an important paper by Skloot et al. [47], also published in 1995, showing that in asthmatics deep inspiration (DI) fails to prevent airway narrowing, suggested a whole new paradigm, the possibility that there might be a fundamental difference in the ASM's response to stress or strain in asthma. A large number of *in vitro* and *in vivo* studies designed to establish the mechanism of this difference followed these publications. Although the physiological processes responsible for the beneficial effects of deep inspiration (DI) are unknown, they are thought to involve mechanical stretch of the ASM during lung inflation [48]. However other factors may also be involved including neural and humoral pathways [49].

### 3.1. Acute Length Perturbations

*In vitro* studies showed that the contractile capability of an isolated ASM strip is attenuated by subjecting it to length oscillations [50, 51]. Fredberg et al. [52] developed a model to demonstrate that mechanical strains in ASM caused by tidal breathing or DI causes detachment of myosin heads from actin sooner than it would during isometric contraction, leading to a steady-state equilibrium. They suggested that in asthma, disruption of this equilibrium leads to the “frozen” contractile state where the muscle is not stretched enough to allow enough mechanical perturbation to disrupt the cross-bridges. 

DI is an inhalation that expands the lung volume toward total lung capacity. There are considerable data showing that DIs are effective in reversing bronchoconstriction in healthy subjects using measurements of resistance (Raw) and forced expiratory volume in one second (FEV_1_). By contrast, DI is not effective or even further exaggerates existing bronchoconstriction in some asthmatic subjects, especially when the airway narrowing occurs during spontaneous or antigen-induced asthmatic attacks [53]. This paradoxical response to DI was recognized as early as the 1960s and 70s [28, 54]. There is a spectrum between the normal response (DI-induced bronchodilation) and severe asthma (DI-induced bronchoconstriction); mild and well controlled asthmatics behave more like nonasthmatics. However it was the observation that DI taken prior to a bronchoconstricting stimulus attenuates the subsequent airway narrowing that has rekindled major interest in this phenomenon [47, 55–61] which has been termed DI-induced bronchoprotection as opposed to bronchodilation. It has been suggested that asthmatics uniquely lack the bronchoprotective effect of DIs [60]. It has long been accepted that stretching contracted ASM by DI reduces bronchospasm by disrupting actin-myosin cross-bridges [52, 62]. However, when DI is taken prior to stimulation, there should be few or no cross-bridges. Hence bronchoprotection could not be explained by a physical detachment of cross-bridges. Wang et al. postulated that the bronchoprotective effect of DI can be explained by the adaptive behavior of ASM in response to DI [48, 51]. 

Length adaptation (also called plasticity) refers to the ability of the muscle to adapt its contractile capacity to length changes as mentioned above. Pratusevich et al. [46] showed that ASM is able to rapidly adapt to different lengths and maintain optimal force generation over a large length range. They observed that the adaptive process consists of two stages, an immediate reduction in force generation following the length change followed by a gradual recovery of the force toward that achieved before the length change. When length oscillation (to simulate DIs) was applied to un-stimulated ASM, a similar two-staged adaptive process were observed [51]. A reduction in active force in response to stimulation was observed immediately after the oscillation and the magnitude of active force reduction was linearly related to the amplitude of the oscillation. After this initial reduction, the muscle undergoes the adaptation process by which active force increases gradually with each stimulation until stabilizing at the level prior to length oscillation. The adaptation process takes about 30 to 40 min to complete depending on animal species and how frequently the muscle is stimulated. McParland et al. [63] showed that ASM from pigs could adapt to a shortened state induced by carbachol within 30 min, resulting in increased force, shortening and shortening velocity.

The two-staged phenomenon in ASM strips after length oscillation resembles the sequence of events during DI-induced bronchoprotection in normal subjects. When DIs are immediately followed by administration of a stimulant, airway luminal narrowing is less than it would be without a DI [64] and the airway resistance is reduced [65]. DI protects the airways from excessive bronchospasm. This is similar to the initial reduced contraction observed in ASM when a length oscillation is applied. However, bronchoprotection by DI is temporary. The stimulant-induced bronchospasm gradually returns to what it would be without a DI. This recovery process is paralleled by the *in vitro* finding that the active force of ASM gradually returns to the same level as prior to length oscillation. These similarities between the bronchoprotection of DI *in vivo* and ASM adaptation *in vitro* suggest that the dynamics of ASM length tension behavior and pathologic alterations in this behavior have the potential to play an important role in airway hyperresponsiveness.

The first evidence for a relationship between length adaptation in ASM and AHR was obtained in guinea pig model of maturation. The recovery of active forcein adult guinea pig ASM is gradual, complete, and follows a time course similar to that observed in the ASM from adults of other species. On the other hand, when length oscillation was applied to ASM obtained from airways of 1-wk old guinea pigs, the subsequent active force increased to about 110% *F*_max⁡_ (*F*_max⁡_: the stable maximal active force generated before mechanical oscillation) and was maintained throughout the adaptation process [66]. This increase of force after the initial reduction was termed force potentiation. These data suggest there is a lack of ASM adaptation in response to mechanical perturbations in immature ASM and is consistent with the clinical observation that airway responsiveness is greater in infants and that DI is ineffective in attenuating airway narrowing in infants as it is in asthmatics [67]. 

More recently Raqeeb et al. [68] studied ASM *in vitro* using dynamic scenarios which more closely resemble *in vivo* airway mechanics where ASM is constantly subjected to low level length oscillations due to tidal breathing interspersed with occasional DI. In their study design they tested the effect of “tidal breathing” with or without “DI” on force development as well as length oscillation in between stimulations during force recovery. They found that adaptation is interrupted by length oscillations, which suggests that in healthy normal lung where ASM is constantly stretched by breathing motions the force could not reach its maximal level, that is, the second stage of adaptation could not be completed. This would be beneficial to maintain airway patency. 

 Most recently, Chin et al. [37] directly compared the effect of length oscillation on tracheal ASM strips from nonasthmatic and asthmatic subjects. Immediately after length oscillation ASM from asthmatics showed less force reduction (~half of that in non-asthmatic ASM) and during subsequent recovery the ASM from asthmatics recovered more rapidly and completely. These results suggest that there is a fundamental difference in ASM response to strain: a reduced response in asthmatics to length oscillations; the difference is intrinsic and not because the strain is reduced by stiffer airways. A reduced initial force reduction is consistent with loss of bronchoprotection in asthmatics.

### 3.2. Subacute/Chronic Length Changes

The effects of subacute and chronic (hours to days) length changes on contractile and structural features have been examined in various skeletal muscle preparations. In the diaphragm, as an example, chronic shortening is clinically relevant in emphysema because the hyperinflation caused by emphysema results in persistent shortening of the muscle. When emphysema is induced in experimental animals by lung elastolysis, the muscle recovers its ability to generate force at short lengths because sarcomeres are subtracted in series over a period of days to weeks [69, 70]. Addition of sarcomeres in series occurs during chronic muscle lengthening [71]. 

Structural remodeling occurs in asthmatic airways over a long time period and includes increased mucus secretion, excessive deposition of extracellular matrix, thickening of the airway wall, smooth muscle cell hypertrophy/hyperplasia and angiogenesis. These structural alterations could influence airway function by causing ASM adaptation to short lengths. Smooth muscle adaptation to prolonged length changes is much faster than skeletal muscle. It was observed that when ASM strips are passively lengthened or shortened *in vitro* over a period of 24 hr, length-tension (*L*-*T*) curves shifted compared to the control curve, allowing maintenance of maximal isometric force at the new length [72]. The result was that smooth muscle adapted to short length was now able to generate the same maximal force as at longer length. Compounding this effect there was a shift of the passive length tension curve to the left, indicating stiffening of the smooth muscle and making reversal of the shortened state more difficult. Naghshin et al. [73] showed that adaptation to passive shortening is reversible after 3 days but not after 7 days. These results suggest that ASM adaptation to shortening can not only occur quickly but also if the shortening conditions persist, more permanent changes can occur. 

Despite the general concordance between *in vitro* and *in vivo* studies and modeling some recent work suggests that simple mechanical explanations for the effects of DI may be simplistic. Transmural pressure changes comparable to those produced by tidal breathing do not affect the response of airway segments to a contractile agonist and with amplitudes greater than 10 cm H_2_O the airways only respond with a transient dilation [74–77]. Noble and colleagues [75] using isolated airway segments also demonstrated that the capacity of simulated deep inspirations to reduce bronchoconstriction is markedly restricted by stiffening of the airway wall in response to contractile stimulation. Furthermore, the transient airway dilation observed in airway segments is smaller, compared to the relatively larger effect seen in intact airways in vivo [78, 79]. 

Additionally some *in vivo* studies are not completely concordant with simple mechanical explanations. Although prior DI has reproducibly been shown to differentially modify the methacholine-induced decline in FEV_1_ in asthmatics and nonasthmatics, other studies have shown that there is no differential effect on the changes in FEV_1_/FVC ratio [80], partial expiratory flow [81] or airway resistance assessed using the forced oscillation technique (FOT) [82]. One interpretation of this discrepancy is that prior DI may not alter the initial airway narrowing produced by a constrictor but instead make the ASM more responsive to subsequent strain during the DI required to perform an FEV_1_ maneuver. This potential mechanism is supported by a recent study of the effect of DI in mice [83].

Together, these data support the idea that the reduced airway response following deep inspiration is likely more complicated than a simple stretch of ASM. One plausible explanation for the ASM response to length oscillation is a corresponding reduction in myosin filament density which has been demonstrated in swine ASM [84]. A similar change in ASM ultrastructure following a DI may explain its bronchoprotective effect. Alterations in myosin filament density in the ASM of asthmatic subjects may make it less prone to disruption following strain, although this has yet to be demonstrated in humans. 

Another potential mechanism which could contribute to AHR which involves ASM is the effect of tone on smooth muscle contractility. As mentioned earlier this is an old idea [25] but has received recent attention due to the studies of Bossé and associates [85–88] who used sheep trachealis to model the effect of basal tone on airway smooth muscle's ability to contract [85]. They have shown that tone induced with cholinergic agonists, prostanoids or histamine results in a more than additive effect on subsequent force production in response to electric field stimulation. They have termed this phenomenon force adaptation and have modeled the increased airway narrowing that could come about because of force adaptation [87]. For example, they calculated that force adaptation occurring in an airway of the 9th generation could increase airway narrowing by 48% and airway resistance by 274%. Force adaption is a very plausible contributor to exaggerated airway narrowing in asthmatics given the abundance of inflammatory mediators and spasmogens that the ASM of asthmatics is exposed to. In addition the loss of this tone when the ASM is examined *in vitro* may be one explanation for the failure of excised asthmatic ASM to show altered contractile function in most studies. A recent study by Pascoe et. al. has tested whether force adaptation could influence ASM function in a setting that is more in line with the *in vivo *environment [86]. In this experiment, ASM strips were subjected to force oscillations that mimicked the forces experienced by the ASM *in vivo* during tidal breathing maneuvers with or without deep inspiration. It was shown that even with force oscillations that mimicked *in vivo* breathing patterns, force adaptation still occurred to the same level as in static conditions. This finding opens the door for future *in vivo* work to explore the role of force adaptation in AHR. It is unlikely that force adaptation is the sole cause of AHR but instead is one of a number of components that leads to AHR in asthmatic subjects. 

In summary, there is a long history of investigation of the role of ASM in airway responsiveness. Despite this extensive research it remains unclear whether a fundamental change in ASM phenotype is the root cause of hyperresponsiveness [88]. Thus, this series of paper in the Journal of Allergy is pertinent and timely. Since airway, hyperresponsiveness is such a fundamental and clinically relevant characteristic of asthmatic airways it is incumbent on us to definitively incriminate or exonerate ASM.
